# A comparative study of bone biopsies from the iliac crest, the tibial bone, and the lumbar spine

**DOI:** 10.1186/s12882-017-0550-5

**Published:** 2017-04-13

**Authors:** Ruth G. G. Hiller, Margret Patecki, Claudia Neunaber, Janin Reifenrath, Jan T. Kielstein, Heike Kielstein

**Affiliations:** 1 Institute for Pathology, University Hospital Halle (Saale), Magdeburger Straße 14, 06112 Halle (Saale), Germany; 2 Department of Nephrology and Hypertension, Hannover Medical School, Hannover, Germany; 3 Trauma Department, Hannover Medical School, Hannover, Germany; 4 Department of Orthopedic Surgery, CrossBIT Center for Biocompatibility and Implant-Immunology, Hannover Medical School, Hannover, Germany; 5 Department of Nephrology and Hypertension, Academic Teaching Hospital Brunswick, Brunswick, Germany; 6 Department of Anatomy and Cell Biology, Martin-Luther-University Halle-Wittenberg, Faculty of Medicine, Halle (Saale), Germany

**Keywords:** Bone biopsy, Trabecular bone volume, Osteoporosis, Renal osteodystrophy, Bone density

## Abstract

**Background:**

Patients with an impaired renal function show a high incidence of bone and mineral disturbances. These ‘chronic kidney disease – mineral and bone disorders’ (CKD-MBD) range from high turnover osteoporosis to adynamic bone disease. Currently, the histomorphometric analysis of a bone biopsy taken from the iliac crest is viewed as the gold standard for CKD-MBD subtype differentiation. However, the clinical relevance of such a biopsy is questionable since iliac crest fractures are an extremely rare finding. Therefore, we aimed to elucidate if the histomorphometric parameter ‘trabecular bone volume (BV/TV)’ from the iliac crest is representative for other biopsy locations. We chose two skeletal sites of higher fracture risk for testing, namely, the tibial bone and the lumbar spine, to examine if the current gold standard of bone biopsy is indeed golden.

**Methods:**

Bone biopsies were taken from 12 embalmed body donors at the iliac crest, the proximal tibia, and the lumbar vertebral body, respectively. Masson-Goldner stained sections of methyl methacrylate embedded biopsies were used for trabecular bone volume calculation. Furthermore, exemplary μ-computed tomography (XtremeCT) scans with subsequent analysis were performed.

**Results:**

Median values of trabecular bone volume were comparable between all body donors with median (interquartile range, IQR) 18.3% (10.9–22.9%) at the iliac crest, 21.5% (9.5–40.1%) at the proximal tibia, and 16.3% (11.4–25.0%) at the lumbar spine. However, single values showed extensive intra-individual variation, which were also confirmed by XtremeCT imaging.

**Conclusions:**

Distinct intra-individual heterogeneity of trabecular bone volume elucidate why a bone biopsy from one site does not necessarily predict patient relevant endpoints like hip or spine fractures. Physicians interpreting bone biopsy results should know this limitation of the current gold standard for CKD-MBD diagnostic, especially, when systemic therapeutic decisions should be based on it.

## Background

Patients with chronic kidney disease have a high risk for bone and mineral diseases caused by changes in calcium and vitamin D metabolism, early menopause, or permanent steroid therapy following renal transplantation [1]*.* Guidelines recommended to base treatment decisions on bone histology due to contrary treatment options for high and low turnover osteopathy [2]. Therefore, trephine bone biopsies usually taken from the iliac crest are the gold standard for the diagnosis of ‘chronic kidney disease – mineral and bone disorders’ (CKD-MBD) [3]. The standard semi-quantitative description of the biopsy specimen gives information about turnover, mineralisation, and bone volume. It is therefore called TMV-classification [4]. Quantitative analysis can be obtained by histomorphometric parameters. For example, osteoporosis is characterized by low trabecular bone volume, which can be represented by the ratio between bone volume and total volume (BV/TV).

Bearing in mind that the iliac crest does not belong to the high risk fracture sites in patients with osteoporosis, it remains uncertain to what extent the histomorphometric data of a biopsy taken from only one skeletal site represents the total burden of a systemic bone disease and can be used for therapeutic decisions.

The aim of our study was to clarify if bone biopsies taken from the iliac crest provide comparable results with other skeletal sites at higher fracture risk such as the vertebral bone or long bones like the tibia [5].

## Methods

The study was approved by the ethics committee of the medical faculty of the Martin Luther University Halle-Wittenberg. All body donors had given written informed consent for scientific investigation prior to death to the Department of Anatomy and Cell Biology in Halle (Saale), Germany as described previously [6].

For the study, we examined 12 human corpses (5 male, 7 female; mean (± SD) age 81.3 (±14.8) years, range 59–99 years). The corpses were preserved after standard embalming techniques, with the embalming solution containing ethanol (77%), unbuffered formalin, glycerine, and distilled water (7% each). From each body donor we extracted a trephine biopsy from the iliac crest (posterior superior iliac spine ‘PSIS’), the proximal tibia (tibial tuberosity) and the 4^th^ vertebral body of the lumbar spine using Jamshidi needles (G7, 10 cm, Gallini S.p.A., Italy). The PSIS and the tibial tuberosity are well defined anatomical land marks and appropriate biopsies could be taken directly through the skin. To ensure taking comparable biopsies from the spine the vertebral bodies were removed from the corpses at first and then bisected in sagittal direction. Biopsies were taken subsequently in the median transverse plane. All biopsies were preserved in 4% formalin solution, dehydrated following standard tissue processing procedures, embedded in methyl methacrylate (Technovit 9100, Heraeus Kulzer, Wehrheim, Germany) and cut in 6 μm thick tissue sections using a sledge microtome (Reichert-Jung, Heidelberg, Germany). Sections were mounted on SuperFrost® microscope slides (Menzel-Gläser, Braunschweig, Germany) and stained with Masson-Goldner.

From each section five randomly selected regions of interest (ROIs) were scanned with the help of a digital microscope (KEYENCE BZ-8000, Mechelen, Belgium) at 100-fold magnification. Following instructions of Egan et al. [7] we used Adobe Photoshop® version CS4 and the image processing program Image J [8] to measure bone area (B.Ar), tissue area (T.Ar) and bone perimeter (B.Pm). Out of the parameters bone area and tissue area the trabecular bone volume (BV/TV) was generated for each sample according to the standardized nomenclature for bone histomorphometry: B.Ar/T.Ar (mm^2^) = BV/TV (%) [9].

High-resolution micro-computed tomography analyses of the plastic embedded bone samples were performed by the use of XtremeCT (Scanco Medical, Zurich, Switzerland) with 60 kVp voltage, 900 μA amperage, an integration time of 300 ms per slice, 1000 projections per 180° and a resolution of 41 μm. Bone sample volume, density and BV/TV were analyzed by the use of a 2-D and true 3-D multi-planar reformatting evaluation software (Evaluation Program V 6.0, Scanco Medial, Zurich, Switzerland) with a defined threshold of 120.

A one way analysis of variance was performed by SPSS program (SPSS Inc. Released 2007. SPSS for Windows, Version 15.0. Chicago, SPSS Inc.) to determine significances between mean values of BV/TV from different biopsy locations; significance level was set at *p* < 0.05. To test relationships of BV/TV ratios between all locations we used Spearman’s correlation coefficient *r*.

## Results

### Median values of trabecular bone volume do not differ significantly between three biopsy sites

Thirty-six bone biopsies were analysed by histomorphometric measurement, as described above. The samples of two body donors had to be excluded from the study because the material from two skeletal sites was not usable. Another set of samples obtaining material from only two of three sites was kept in the study.

**Fig. 1:**
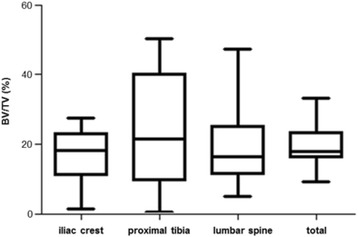
Median values of BV/TV at three biopsy sites. Trabecular bone volume (BV/TV) was generated out of bone area and tissue area measurements for each biopsy site. Median values and data distribution were outlined for iliac crest, proximal tibia and lumbar spine separately, and as “total” for mean BV/TV values from all biopsy sites

From the remaining 29 samples, trabecular bone volume (BV/TV) was analysed. Figure 1 shows the median trabecular bone volume of each biopsy site separately and the overall mean value. At the iliac crest the median BV/TV (IQR; mean ± SD) was 18.3% (10.9–22.9%; 16.4 ± 8.3%), at the proximal tibia 21.5% (9.5–40.1%; 24.4 ± 17.1%), and at the lumbar spine it was 16.3% (11.4–25.0%; 19.6% ± 12.4%).

Table 1 shows the correlation between the BV/TV values from the three sampling sites. There was no significant correlation found between the BV/TV ratios of the three examined sampling sites.

**Table 1:** Correlation between BV/TV measurements at three biopsy sides

BV/TV (%)		iliac crest	proximal tibia	lumbar spine
iliac crest	Spearman correlation (r) significance (2-side) (p) N	1 ᅟ 10	−0.04 0.907 10	−0.37 0.332 9
proximal tibia	Spearman correlation (r) significance (2-side) (p) N	−0.04 0.907 10	1 ᅟ 10	−0.35 0.356 9
lumbar spine	Spearman correlation (r) significance (2-side) (p) N	−0.37 0.332 9	−0.35 0.356 9	1 ᅟ 9

Spearman correlation was calculated between trabecular bone volume values from all three biopsy sites. *P*-values < 0.05 were stated significant

### Trabecular bone volume shows a high intra-individual diversity

**Fig. 2:**
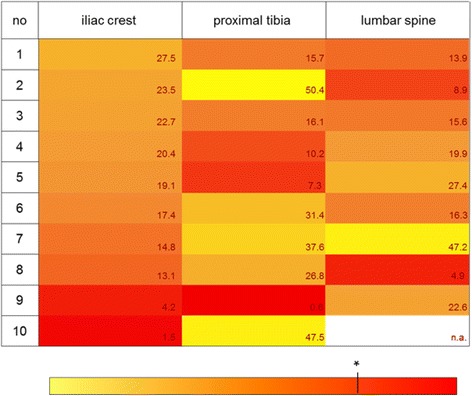
Intra-individual differences between BV/TV values. BV/TV ratios were colour-coded in a heat map manner. The colour-bar below describes the BV/TV values, with yellow colour representing high values and red colour representing lower values. * The asterisk marks a BV/TV ratio of 10 which is associated with an apparent osteoporotic bone. The BV/TV results were illustrated for each biopsy site separately. The results from each donor were ordered top down by size of the BV/TV ratio from the standard biopsy site iliac crest

Median trabecular bone volume slightly differed between the biopsy sites. However, to use information from an iliac crest biopsy as a surrogate for diagnostic and therapeutic decisions presumably with systemic impact we wanted to validate our findings for each body donor. Therefore, individual BV/TV measurements were analysed. To visualize possible heterogeneity the results were colour-coded according to their values in a heat map manner. Figure 2 shows BV/TV measurements after colour coding for all body donors and all biopsy sites. The body donors were sorted by the BV/TV results from iliac crest top-down beginning with the highest trabecular bone volume result. In only 2 of the 10 cases (no. 4 and no.6) the iliac crest BV/TV represented at least one of the other sites (lumbar spine). Apart from that, it is clearly visible that there were great intraindividual differences between the sampling sites. For nearly all sample sets the BV/TV of the iliac crest overestimated BV/TV from at least one of the other biopsy sites. For donor number 2, 5, and 8 the BV/TV difference was so high that severe osteoporosis at either proximal tibia (no. 5) or lumbar spine (no’s. 2, 8) would not have been reflected by the iliac crest biopsy result. On the other hand, low iliac crest BV/TV of number 7 could not be confirmed by any other site.

### Histomorphometric BV/TV results are reproducible by XtremeCT imagine

To confirm the histological bone trabecular volume measurements we performed XtremeCT imaging in selected specimens that showed either comparable BV/TV results or markedly divergent BV/TV results at the sample sites. The results are shown in Table 2. For sample set number 3 similar BV/TV results at proximal tibia and lumbar spine were comparable with XtremeCT imaging. In sample set number 8 both methods comparably reveal the large difference between BV/TV at the respective biopsy sites albeit with greater deviation between the ratios.

**Table 2:**
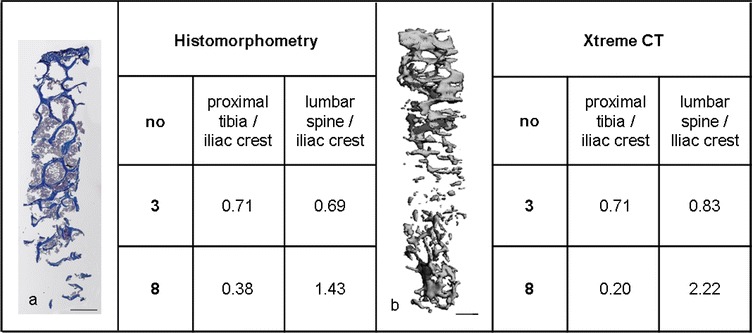
Comparison between BV/TV results from histomorphometry and XtremeCT

BV/TV values of two donors (no.3 and no.8) were obtained by histomorphometry and XtremeCT imaging. BV/TV results of proximal tibia and lumbar spine were related to correspondent iliac crest results and listed as ratios. Exemplary bone biopsy images from histology (a, scale bar = 1 mm) and XtremeCT (b, scale bar = 1 mm) are shown, in addition

## Discussion

‘Chronic kidney disease - mineral and bone disorders’ (CKD-MBD) are diseases, which can be subdivided by bone histological parameters of bone turnover (T), mineralisation (M) and bone volume (V) [4]. For example, osteoporosis as a frequent disease with high fracture risk is presented by reduced bone volume, whereas mineralisation and turnover can be altered in different directions. Therefore, current guidelines recommend a bone biopsy before induction of treatment [2]. The therapeutic goal is the prevention of patient relevant endpoints, especially fractures. In patients on dialysis the prevalence of fractures is four times higher and they occur about 10–15 years earlier than in the general population. Furthermore, these patients have a dramatically elevated 1-year mortality risk after fracture [10]. Unfortunately, it is not clear how to predict fracture risk in CKD patients. In the general population bone density measurement by dual x-ray absorptiometry (DXA) is the gold standard for osteoporosis diagnostics and fracture risk prediction. While osteoporosis is clearly one major contributor to the elevated fracture risk of the CKD population DXA was not recommended for CKD patients so far [2]. One reason was that DXA cannot predict the CKD-MBD subtype. However, recent studies show that DXA can nevertheless predict fracture risk of patients with chronic kidney disease [11] and will presumably be an inherent part of the CKD-MBD diagnosis in the future.

Concerning bone biopsies there is uncertainty whether an improvement in the bone biopsy parameters, which is often used to show treatment effects, results in reduced fracture risk or not. For example, in the Bonafide study the treatment with the calcimimetic drug cinacalcet resulted in a normalisation of elevated bone turnover but has no effect on the bone volume [12]. Nevertheless, in another trial with cinacalcet, a reduction of fracture risk was shown, even if this was only pronounced when adjusting to baseline differences [13].

In the present study bone specimens of embalmed cadavers were used for the histomorphometric assessment. The median bone volume of all 10 body donors was comparable between the three biopsy sites (Fig. 1) but revealed severe intraindividual differences (Fig. 2). Only a few biopsy studies investigated bones from two or more skeletal sites. Their results are very heterogeneous. While some groups showed a more or less pronounced correlation [14] other studies could not confirm this [15] or showed different results depending on the sites [16]. Biopsy studies including histomorphometric measurements from the human tibia are not known so far whereas the proximal tibia is one of the common investigation sites for bone diseases in rats. In animal studies the iliac bone structure and metabolism showed an average between those of the tibia and the lumbar vertebra [17]. Those findings could not be confirmed in our human samples where no relation could be seen between the biopsy sites at all.

High-resolution imaging methods can determine bone strength and quality in-vivo. Furthermore, they can be used for bone biopsy analyses. For example, Borah and colleagues used micro-computed tomography of bone biopsies to describe effects of long-term bisphosphonate treatment on BV/TV results [18]. In our study, we wanted to validate our histomorphometric findings by micro-computed XtremeCT-analysis of the same specimen. For this, we chose two sample sets that revealed very different results in the histomorphometric analysis. For donor number 3 the BV/TV-ratio between the proximal tibia and the iliac crest were comparable with the ratio between the lumbar spine and the iliac crest. In contrast, donor number 8 was chosen for the great difference between those BV/TV ratios. By confirming our histomorphometric results with the imaging method (Table 2), we could exclude that the measured BV/TV-heterogeneity was due to method-derived failures.

The intra-individual differences seen in our study may reflect the variability of skeletal sites due to requirements of each individual and the anatomic position. The skeleton shows a region-specific difference of the two osseous tissue types, i.e. trabecular and cortical bone. Depending on the anatomic site, these two bone types exhibit distinct microstructure characteristics and mechanical properties that, furthermore, differ between the anatomic sites [19]. In our study we used bone biopsy samples from three skeletal regions with different requirements of strength and consecutively specific amounts of trabecular and cortical bone. For example, the vertebral body mainly consists of trabecular bone, whereas the tibia as a long bone of the lower limbs is predominantly built up of cortical bone [20].

Trabecular bone is composed of irregularly distributed rods and plates [21] and the cortical bone consists of regular, cylindrically shaped lamellae [19]. Due to diverse directions of these structures, which is called the anisotropy of the bone, the mechanical properties of the bone are very variable. Thus, cortical bone has directional properties in pressure and traction strength, as well as in elastic modulus, that could be confirmed in bone specimen analysis which were sampled either in longitudinal or transverse direction [22].

Another explanation of the intra-individual variability of bone volume is the different level of mechanical load at each anatomic region, which results in either weakening or strengthening of the bone. Thus, the dominant hand becomes usually stronger with growth leading to a limb bilateral asymmetry [23] and one-sited exercise, e.g. tennis, can result in a higher bone mineral content at this site [24].

In addition, CKD-MBD have an impact on the two bone types. For example, early hyperparathyroidism leads to a reduced bone volume pronounced at the cortical sites [25]. Unfortunately, clinical information about the renal function from the body donors was not available in our study. On the other hand, the mean age of the body donors was 81 years where an average kidney function below GFR 60 ml/min and therefore early changes in mineral and bone metabolism could be supposed [2]. Nevertheless, even if we cannot directly transfer the results of this study into the CKD population, the shown limitation of the bone biopsy should create awareness and encourage further studies.

In summary, our data show that trabecular bone volume (BV/TV) results of one skeletal site are not always representative of that of other sites, hence it is no *pars pro toto*. The heterogeneity of the skeleton must be known by the physician interpreting bone biopsy results. Thomson et al. suggest that taking a biopsy from the site of a fracture might deliver the patient relevant information about the bone metabolism and structure [26]. Unfortunately, this might not be practicable in the clinical setting and taking biopsies from skeletal regions at higher fracture risks like the lumbar spine is not feasible. Therefore, we consider using imaging measurements, e.g. bone mineral density results, which can be taken at several skeletal sites for an individual fracture risk assessment.

Future studies will have to evaluate our results in the CKD population and whether parameters of mineralisation and turnover show the same intraindividual heterogeneity compared to bone volume.

## Conclusion

Bone biopsies are the gold standard for the diagnosis of CKD-MBD providing information about bone volume, mineralisation, and bone turnover. Histomorphometric biopsy results from different skeletal sites show a high intraindividual diversity, at least for bone volume. This advises caution for drawing general conclusions from a one sited bone biopsy. Additional diagnostic tools might be necessary to describe skeletal heterogeneity.
